# Neurophysics: Understanding brain activity with modeling complex systems mathematics

**DOI:** 10.1016/j.clinsp.2022.100158

**Published:** 2023-01-21

**Authors:** Fulvio A. Scorza, Ana C. Fiorini, Antônio M. Rodrigues, Carla A. Scorza, Gabriel D. Vilallonga, Marcelo A. Moret, Tarcísio M. Rocha Filho, Josef Finsterer, Antônio-Carlos G. de Almeida

**Affiliations:** a Disciplina de Neurociência, Escola Paulista de Medicina, Universidade Federal de São Paulo (EPM/UNIFESP), São Paulo, SP, Brazil; b Centro de Neurofísica “Professor Hiss Martins-Ferreira”, Escola Paulista de Medicina, Universidade Federal de São Paulo (EPM/UNIFESP), São Paulo, SP, Brazil; c Departamento de Fonoaudiologia, Escola Paulista de Medicina, Universidade Federal de São Paulo (EPM/UNIFESP), São Paulo, SP, Brazil; d Programa de Estudos Pós-Graduado em Fonoaudiologia, Pontifícia Universidade Católica de São Paulo (PUC-SP), São Paulo, SP, Brazil; e Laboratório de Neurociência Experimental e Computacional, Departamento de Engenharia de Biossistemas, Universidade Federal de São João del-Rei (UFSJ), São João del-Rei, MG, Brazil; f Universidad Nacional de Catamarca, Argentina; g SENAI-Cimatec, Salvador, BA, Brazil; h Departamento de Ciências Exatas e da Terra (DCET), Universidade do Estado da Bahia (UNEB), Salvador, BA, Brazil; i International Center for Condensed Matter Physics, Universidade de Brasília, Brasília, DF, Brazil; j Instituto de Física, Universidade de Brasília, Brasília, DF, Brazil; k Neurology & Neurophysiology Center, Vienna, Austria

Science is always dynamic. New discoveries in health sciences are used to detect, treat, and prevent disease, that not only improve our quality of life, but extend our lifespans. In this scenario, translational studies play a key role. Translational research has been recognized as the result of the continuous scientific advances, which can be divided into two areas. The first involves applying insights from basic science to the development of human studies. These analyses are often performed using animal models, cultures of tissues, human and animal cell samples, ranging from molecular aspects to modeling complex systems mathematics. The second translational domain brings together the results of these basic studies and their applicability in clinical practice. In particular, in neuroscience, the area of biomedical sciences most prominent in industrialized countries, there is a need to underpin the models of a theoretical and computational approach with physical knowledge to understand biological processes. Basically, neurophysics is an interdisciplinary science that uses physics and combines it with other neurosciences to understand the neural processes.

For more than a decade, our research group has been developing neurophysical studies using different approaches. Based on computer simulations, we have shown that induction and maintenance of epilepsy occurs through non-synaptic activity.^1^ We sequentially showed that ethanol consumption promotes changes on the non-synaptic mechanisms that modulate non-synaptic epileptiform activities. Interestingly, the neurophysiological changes were more effective in the lower ethanol dosage, suggesting that they are due to the less intense neurodegeneration.^2^ In 2015, we verified how non-synaptic epileptiform activity in the hippocampal formation were affected by kainic acid administration and showed that elements involved in non-synaptic events are potential targets for drugs to treat neurological disorders where intrinsic excitability is a problem.^3^ We then proposed simple rules for learning, inspired by recent machine learning insights and adapted to a realistic spiking neural network, and showed that the incorporation of non-synaptic interaction mechanisms improves cell assembly convergence.^4^ In 2020, our group first proposed how Computational Neuroscience (CN) offers the possibility to prevent Sudden Unexpected Death in Epilepsy (SUDEP), leading to better insight into triggering mechanisms.^5^ Recently, we presented an epidemiological model, developed from real past data to verify the effectiveness of different strategies of vaccination against COVID-19 and in particular the role of individuals with a significantly higher number of contacts in the spread of the virus.^6^

From these data and perspectives, it can be seen that the physical bases of the most relevant neurological diseases (epilepsies, Alzheimer's disease and other dementias, cerebrovascular disease, Central Nervous System [CNS] tumors, Parkinson's and diseases of the motor system, in addition to retinopathies and inflammatory and infectious diseases of the CNS) needs to be further explored in the light of a rigorous mathematical approach.

By analyzing such evidence and setting an agenda for the future, our group research aims to provide a way to take the necessary actions to improve population health. Thus, one of the central tasks of our Neurophysics Center “Professor Hiss Martins-Ferreira” will contribute to intensify the generation of knowledge and the training of human resources in neurophysics with greater international reach. Therefore, our center's proposal to support national and international groups of excellence in neurophysics will contribute significantly to the implementation of computational approaches to the nervous system, to train qualified human resources and to generate scientific and technological knowledge. In addition, the strengthening of the groups of our neurophysics center “Professor Hiss Martins-Ferreira” also has an impact on the qualification of a large number of research groups employees.

Overall, neuroscience research is related to many fields of discipline. These multidisciplinary fields affect not only the human health but also computacional science, i.e., Neurophysics. Finally, it is important to emphasize the words of Carl Sagan: “We've arranged a civilization in which most crucial elements profoundly depend on science and technology.”

## Conflicts of interest

The authors declare no conflicts of interest.
